# Mental health impacts among health workers during COVID-19 in a low resource setting: a cross-sectional survey from Nepal

**DOI:** 10.1186/s12992-020-00621-z

**Published:** 2020-09-25

**Authors:** Pratik Khanal, Navin Devkota, Minakshi Dahal, Kiran Paudel, Devavrat Joshi

**Affiliations:** 1 Institute of Medicine, Tribhuvan University, Kathmandu, Nepal; 2 National Academy for Medical Sciences, Kathmandu, Nepal; 3 Center for Research on Environment, Health and Population Activities (CREHPA), Kathmandu, Nepal

**Keywords:** Anxiety, COVID-19, Depression, Health workers, Insomnia, Mental health, Nepal

## Abstract

**Background:**

Health care workers exposed to COVID-19 might be at increased risk of developing mental health problems. The study aimed to identify factors associated with anxiety, depression and insomnia among health workers involved in COVID-19 response in Nepal.

**Methods:**

This was a cross-sectional web-based survey conducted between April 26 and May 12, 2020. A total of 475 health workers participated in the study. Anxiety and depression were measured using a 14-item Hospital Anxiety and Depression Scale (HADS: 0–21) and insomnia was measured by using a 7-item Insomnia Severity Index (ISI: 0–28). Multivariable logistic regression analysis was done to determine the risk factors of mental health outcomes.

**Results:**

Overall, 41.9% of health workers had symptoms of anxiety, 37.5% had depression symptoms and 33.9% had symptoms of insomnia. Stigma faced by health workers was significantly associated with higher odds of experiencing symptoms of anxiety (AOR: 2.47; 95% CI: 1.62–3.76), depression (AOR: 2.05; 95% CI: 1.34–3.11) and insomnia (AOR: 2.37; 95% CI: 1.46–3.84). History of medication for mental health problems was significantly associated with a higher likelihood of experiencing symptoms of anxiety (AOR: 3.40; 95% CI:1.31–8.81), depression (AOR: 3.83; 95% CI: 1.45–10.14) and insomnia (AOR: 3.82; 95% CI: 1.52–9.62) while inadequate precautionary measures in the workplace was significantly associated with higher odds of exhibiting symptoms of anxiety (AOR: 1.89; 95% CI: 1.12–3.19) and depression (AOR: 1.97; 95% CI: 1.16–3.37). Nurses (AOR: 2.33; 95% CI: 1.21–4.47) were significantly more likely to experience anxiety symptoms than other health workers.

**Conclusion:**

The study findings revealed a considerate proportion of anxiety, depression and insomnia symptoms among health workers during the early phase of the pandemic in Nepal. Health workers facing stigma, those with history of medication for mental health problems, and those reporting inadequate precautionary measures in their workplace were more at risk of developing mental health outcomes. A focus on improving mental wellbeing of health workers should be immediately initiated with attention to reduction of stigma, ensuring an adequate support system such as personal protective equipments, and family support for those with history of mental health problems.

## Background

The new coronavirus disease 2019 (COVID-19) is currently a threat to the global health in an unprecedented manner. Nepal, a South Asian country, is no exception and is affected by the outbreak with overwhelming effects on its economy and health system. The Government of Nepal initiated its response against COVID-19 immediately after its first reported case in the last week of January in a Nepalese traveller from China [1, 2]. As of June 29, 13,248 cases and 29 deaths had been reported in the country despite nationwide lockdown imposed from March 24, which continued for nearly 10 weeks [3].

The mental health impact of a disease outbreak is usually neglected during pandemic management although the consequences are costly [4]. Early evidence has shown that health workers directly involved in the diagnosis, treatment, and care of patients with COVID-19 are at risk of developing mental health symptoms [5–8]. Similar adverse psychological reactions were reported among health care workers in previous studies during the 2003 Severe Acute Respiratory Syndrome (SARS) outbreak [9, 10]. The increasing number of confirmed cases and deaths, work burden, inadequate personal protective equipment (PPE), media coverage, lack of specific treatment, vulnerability to infection and having to stay in quarantine, as well as feelings of being inadequately supported in the workplace, can contribute to the mental burden of health workers [11].

Psychological wellbeing has an important impact on individuals’ performance. The impact of COVID-19 on mental health is well documented in various countries among different populations including health professionals [4]. However, evidence regarding the impact of the COVID-19 pandemic on health professionals is not available in Nepal. During the initial response to COVID-19, there were media reports regarding inadequate testing kits, and lack of PPEs [12, 13]. At the work place, health workers require a support system to promote their mental wellbeing and their activity need to be continually monitored - this is crucial during health emergencies [14]. A timely assessment of mental health status and mental health needs of health workers during emergencies will help the management to respond and reduce psychological distress, and also align health workers to the patient needs. In this context, this study aimed to evaluate mental health outcomes among health care workers involved in the COVID-19 response by quantifying the magnitude of symptoms of depression, anxiety and insomnia and by analysing potential risk factors associated with these symptoms.

## Methods

### Study design and study participants

A cross-sectional study was conducted via web online survey among health workers working in health facilities in Nepal. Data were collected from April 26 to May 122,020. During the data collection period, Nepal experienced an increase in COVID-19 reported cases from 52 to 217. As of May 12, cases were reported from 19 out of 77 districts of Nepal.

Health professionals working in the management of COVID-19 response, in both public and private health facilities were recruited as study participants through online survey. A network of 25 hub hospitals are designated for COVID-19 management while other hospitals, primary health centres and health posts coordinate with these hub hospitals and run fever clinics for screening of COVID-19 cases. Health professionals included doctors, nurses, pharmacists, diagnostic personnel, paramedics and public health practitioners. A total of 501 responses were received out of which 26 were redundant and hence removed from the analysis. The final sample size of the study was 475.

### Data collection methods

The data collection involved two steps: i) identification of survey anchors for participant recruitment online, and ii) survey administration. In the first step, we identified social media platforms and health facility focal persons to recruit the participants followed by non-random sampling of participants interested to participate in the online survey. Online questionnaires on online Google forms were used to collect data from the participants. Study participants were encouraged to fill the online survey form in their leisure. To limit non-health worker’s responses to the online survey, forms were only sent upon invitation to potential participants. The inclusion criteria were health workers aged 18 years and above and living in Nepal, and currently working in COVID-19 management. Participants were excluded if they were below 18 years of age, on leave or unable to participate due to physical or emotional distress.

### Study variables

The dependent variables in the study included the status of anxiety, depression and insomnia. The independent variables included information about socio-demographic characteristics and work-related variables. The dependent variables and independent variables are presented in Table 1.

**Table 1:** Study variables

S.N.	Variables	Categories of variables
Dependent variables		
**1**	Anxiety	Normal (0–7) and Anxiety (more than 7) based on Hospital Anxiety and Depression Scale
**2**	Depression	Normal (0–7) and Depression (more than 7) based on Hospital Anxiety and Depression Scale
**3**	Insomnia	No clinically significant insomnia (0–7), Sub threshold insomnia (8–14), Moderate severity (15–21) and Severe clinical insomnia (22–28) based on Insomnia Severity Index. For analysis purpose, a cut-off score of 10 was taken. Absence (0–9) and Presence of insomnia (10 and above)
Independent variables		
Socio-demographic characteristics		
**1**	Age	Up to 40 years, Above 40 years
**2**	Gender	Male, Female
**3**	Ethnicity	Brahmin/Chhetri, Janajati, Madheshi and others. Adopted from Nepal’s Health Management Information System
**4**	Educational qualification	Intermediate and below, Bachelor, Masters and above
**5**	Profession	Doctors, Nurses, Others
**6**	Marital status	Single, Ever married
**7**	Type of family	Nuclear, Joint and Extended
**8**	Living with child less than 15 years	Yes, No
**9**	Living with elderly (above 60 years)	Yes, No
**10**	Having a family member with chronic disease	Yes, No
**11**	History of medication for mental health problem	Yes, No
Work related variables		
**1**	Work role	Frontline, second line
**2**	Work experience	Up to 5 years, more than 5 years
**3**	Type of health facility	Primary, secondary and tertiary
**4**	Precautionary measures in workplace	Sufficient, insufficient
**5**	Aware of government incentive for health workers	Yes, No
**6**	Stigma faced due to COVID-19	Yes, No, Do not want to answer
**7**	Working in affected district	Yes, No (District having at least one confirmed case as affected district)
**8**	Working overtime	Yes, No
**9**	Change in regular job duty	Yes, No

### Data collection measures

Anxiety, depression and insomnia of the participants were assessed using the 14-item Hospital Anxiety and Depression Scale (HADS), and the 7-item Insomnia Severity Index (ISI). Internal consistency of the tool was ascertained by calculating Cronbach’s alpha, which was 0.81, 0.72 and 0.90 for anxiety, depression and insomnia respectively and considered sufficient [15, 16].

The HADS is a commonly used tool for measuring anxiety and depression in different settings in many countries including Nepal [17–22]. It has seven items each for measurement of anxiety and depression which are scored from 0 to 21. The total scores of these tools were interpreted as normal (0–7), borderline abnormal (8–10) and abnormal (11–21). For analysis, score more than 7 was considered as the presence of anxiety and depression. Similarly, the score of ISI which records sleep outcome in the past 2 weeks was categorised as no clinically significant insomnia (0–7), subthreshold insomnia (8–14), moderate clinical insomnia (15–21) and severe clinical insomnia (22–28) as in studies done elsewhere [8, 23–26]. For further analysis, a cut-off score of 10 was used to categorise the presence or absence of insomnia as suggested by Morin CM et al. [27].

### Data analysis

Descriptive analysis was done by calculating frequency and percentages for categorical variables and mean and standard deviation for continuous variables. Chi-square test was used to determine the association between categorical independent variables and categorical dependent variables (Additional file 1). To determine potential factors associated with the outcome variable, multivariable logistic regression analysis was performed, adjusted odds ratio (AOR) and 95% confidence interval (CI) were calculated. For adjusted regression analysis, those variables which were significant at a 10% significance level in bivariate analysis were included in the multivariable logistic regression analysis [28]. Similarly, the history of medication for a mental health problem was also fitted into the model regardless of the significance based on the prior knowledge [29]. The Variance Inflation Factor (VIF) was calculated before fitting into the model for each of the psychometric scales which showed no evidence of multicollinearity (less than 1.3).

In the multivariable logistic regression models, the effect of gender, ethnicity, profession, education, living with elderly, family member with chronic disease, precautionary measures in the workplace, faced stigma, worked overtime, awareness about government incentive and history of medication for mental health problem was adjusted to identify the factors associated with anxiety symptoms. Similarly for depression, the effect of age, ethnicity, profession, education, living with children, precautionary measures in the workplace, faced stigma, awareness about government incentive and history of medication for mental health problem was adjusted. Likewise for insomnia, the effect of age, ethnicity, profession, education, work experience, living in affected district, faced stigma, working overtime, awareness about government incentive and history of medication for mental health problem was adjusted.

### Ethics

Ethical approval for the study was given by the Nepal Health Research Council (Reference number: 2192, 315/2020). Written digital consent was taken from study participants prior to completing the survey form. Participants gave their consent by ticking the designated box. Personal identifiers such as name were not collected during the study. The email address collected from the study participants was only used for quality control and not for analysis purposes.

## Results

### Socio-demographic characteristic of study participants

Of the study participants, 52.6% were female, 68.4% were in the age group of 20–29 years and 65.9% belonged to the Brahmin/Chhetri ethnic group. The mean (±SD) age of the participants was 28.20 (±5.80) years. More than two-thirds of health workers were either nurses (35.2%) or doctors (33.9%). The majority of the participants were single (62.9%) and had a nuclear family structure (64.8%). More than half of the participants (54.5%) had a family member with a chronic disease condition, 25.1% were living with younger children and 34.3% had elderly people in the family. The percentage of health workers who had a history of medication for any kind of mental health conditions was 4.6% (Table 2).

**Table 2:** Socio-demographic characteristics of study participants (*n* = 475)

Variables	Categories	Number	Percentage
Age (years)	Mean(±SD): 28.20 (±5.80)		
	20–29	325	68.4
	30–39	124	26.1
	40–49	19	4.0
	50 and above	7	1.5
Sex	Male	225	47.4
	Female	250	52.6
Ethnicity	Brahmin/Chhetri	313	65.9
	Janjati	110	23.2
	Madhesi	29	6.1
	Dalit	7	1.5
	Others	16	3.4
Education	Technical school level	8	1.7
	Intermediate	86	18.1
	Bachelors	277	58.3
	Masters and above	104	21.9
Position	Nurse	167	35.2
	Doctor	161	33.9
	Paramedics	81	17.1
	Laboratory staff	19	4.0
	Pharmacist	15	3.2
	Public health professional	32	6.7
Marital status	Single	299	62.9
	Ever married	176	37.1
Family type	Nuclear	308	64.8
	Joint	167	35.2
Living with children	Yes	119	25.1
	No	356	74.9
Living with older adults (> 60 years)	Yes	163	34.3
	No	312	65.7
Family member with a chronic medical condition	Yes	259	54.5
	No	216	45.5
History of medication for mental health	Yes	22	4.6
	No	453	95.4

### Work-related characteristics

Regarding the type of health facility, 39% worked in a central or provincial hospital, and 28.2% worked in a private hospital. Nearly half of the participants (45.3%) mentioned working as frontline workers for COVID management while 70.7% had started their job within the last 5 years. The majority of the participants reported changes in their regular job duties (70.3%) and insufficient precautionary measure in their workplace (78.9%) during the outbreak. Around half of the participants (49.1%) were working overtime. The proportion of health workers aware of government incentive scheme for health workers during COVID-19 was 56.8%, of which 69.6% were dissatisfied with this scheme. More than half of the participants (53.7%) faced stigma from the community members. Among those who faced stigma, they were stigmatised because of profession (49.8%), accused of being a carrier of the disease (40.0%), threatened (5.9%) or asked to leave their rented place (4.3%) (Table 3).

**Table 3:** Work-related characteristics of the study participants (*n* = 475)

Variables	Category	Number	Percentage
Level of health institution	Central hospital and province hospital	185	39.0
	Private hospital	134	28.2
	Health post	45	9.5
	Community hospital	27	5.7
	Primary hospital (under local government)	20	4.2
	Primary Health centre	19	4.0
	Managerial task of COVID19	45	9.5
Type of health facility	Primary	84	17.7
	Secondary and tertiary	391	82.3
Work role	Front line	215	45.3
	Second line	260	54.7
Working experience (years)	Up to 5	336	70.7
	> 5	139	29.3
Precautionary measures in the workplace	Sufficient	100	21.1
	Not sufficient	375	78.9
Experience of stigma due to occupation	Yes	255	53.7
	No	199	41.9
	Don’t want to answer	21	4.4
Type of major stigma experience (*n* = 255)	Stigmatised because of profession	127	49.8
	Accused of being a carrier of disease	102	40.0
	Threatened	15	5.9
	Asked to leave rented place	11	4.3
Aware of government incentives for health workers	Yes	270	56.8
	No	205	43.2
Satisfied with government incentive (*n* = 270)	Yes	82	30.4
	No	188	69.6
Change in regular job duties during covid19	Yes	334	70.3
	No	141	29.7
Working overtime during COVID-19	Yes	233	49.1
	No	242	50.9

### Prevalence of anxiety, depression and insomnia

More than one-third of the participants had some symptoms of anxiety (borderline: 23.6% and abnormal: 18.3%). Similarly, 37.5% of the participants experienced symptoms of depression (borderline: 24% and abnormal: 13.5%). Likewise, symptoms of insomnia were prevalent in 33.9% of the participants (sub-threshold insomnia: 26.7%, moderate insomnia: 5.7% and severe clinical insomnia: 1.5%).There was a significant difference in anxiety (*p* < 0.001) and depression (*p* = 0.001) across different types of profession. However, type of profession was not statistically significant with insomnia (*p* = 0.142). Nurses had a higher proportion of symptoms related to abnormal anxiety, abnormal depression and severe clinical insomnia than other professions (Table 4).

**Table 4:** Prevalence of anxiety, depression and insomnia by study groups (*n* = 475)

Mental health outcomes	Categories	Total N (%)	Doctor (*n* = 161)	Nurse (*n* = 167)	Other health workers (*n* = 147)	*P*-value *
Anxiety	Normal	276 (58.1)	106 (65.4)	73 (43.7)	97 (66.4)	< 0.001
	Borderline	112 (23.6)	34 (21.0)	54 (32.3)	24 (16.4)	
	Abnormal	87 (18.3)	22 (13.6)	40 (24.0)	25 (17.1)	
Depression	Normal	297 (62.5)	122 (75.3)	89 (53.3)	86 (58.9)	0.001
	Borderline	114 (24.0)	27 (16.7)	46 (27.5)	41 (28.1)	
	Abnormal	64 (13.5)	13 (8.0)	32 (19.2)	19 (13.0)	
Insomnia	No clinically significant	314 (66.1)	115 (71.0)	98 (58.7)	101 (69.2)	0.142
	Sub threshold	127 (26.7)	38 (23.5)	53 (31.7)	36 (24.7)	
	Moderate	27 (5.7)	9 (5.6)	12 (7.2)	6 (4.1)	
	Severe	7 (1.5)	0 (0)	4 (2.4)	3 (2.1)	

*Significant at *p* < 0.05, Chi-square test

### Factors associated with anxiety, depression and insomnia among health workers

Stigma experience among health workers was significantly associated with higher odds of experiencing symptoms of anxiety (AOR: 2.47; 95% CI: 1.62–3.76), depression (AOR: 2.05; 95% CI: 1.34–3.11) and insomnia (AOR: 2.37; 95% CI: 1.46–3.84). History of medication for mental health problems was significantly associated with higher likelihood of experiencing symptoms of anxiety (AOR: 3.40; 95% CI: 1.31–8.81), depression (AOR: 3.83; 95% CI: 1.45–10.14) and insomnia (AOR: 3.82; 95% CI: 1.52–9.62). Inadequate precautionary measures in the workplace was significantly associated with higher odds of exhibiting symptoms of anxiety (AOR: 1.89; 95% CI: 1.12–3.19) and depression (AOR: 1.97; 95% CI: 1.16–3.37). As compared to *Brahmin/Chhetri* ethnic group, *Janajati* had significantly higher odds of having symptoms of anxiety (AOR = 2.34; 95% CI: 1.44–3.81) and insomnia (AOR = 1.74; 95% CI: 1.04–2.91). Profession wise, nurses (AOR = 2.33; 1.21–4.47) had significantly higher odds of having anxiety symptoms than other health workers while doctors (AOR = 0.57; 95% CI: 0.33–0.99) had significantly lower odds of experiencing symptoms of depression than other health workers. Younger health workers (AOR = 0.33; 95% CI: 0.12–0.91) and those aware of the government incentive for health workers during COVID-19 (AOR = 0.51; 95% CI: 0.34–0.78) were significantly less likely to exhibit symptoms of depression compared with older health workers and those not aware of such incentives. Regarding work experience, those who had less than 5 years’ work experience (AOR = 0.50; 95% CI: 0.29–0.85) had lower odds of having symptoms of insomnia compared with those with experience of more than 5 years. Gender, education, living with elderly people, a family member with chronic disease, working overtime and awareness about government incentive was however not statistically significant with the presence of anxiety symptoms. Similarly, ethnicity, education and living with children were not statistically significant with the presence of depression symptoms. Likewise, age, profession, education, working in affected district, working overtime and awareness about government incentive was not statistically significant with the symptoms of insomnia (Tables 5, 6 and 7).

**Table 5:** Factors associated with anxiety among health workers (*n* = 475)

Variables	Category	Anxiety N (%)	Unadjusted OR (95% CI)	Adjusted OR (95% CI)
Gender	Male	76 (38.2)	Ref	Ref
	Female	123 (61.2)	1.90 (1.31–2.75)*	1.05 (0.59–1.88)
Ethnicity	Brahmin/Chhetri	110	Ref	Ref
	Janajati	69	2.71 (1.75–4.20)*	2.34 (1.44–3.81)*
	Madheshi	10	0.97 (0.44–2.16)	1.11 (0.47–2.59)
	Others	10	2.64 (0.98–7.12)	2.19 (0.73–6.54)
Profession	Doctor	56	1.05 (0.65–1.68)	1.17 (0.68–2.04)
	Nurses	94	2.55 (1.61–4.04)*	2.33 (1.21–4.47)*
	Others	49	Ref	Ref
Education	Intermediate and below	47	Ref	Ref
	Bachelor	116	0.72 (0.45–1.15)	0.97 (0.57–1.67)
	Masters and above	36	0.53 (0.30–0.94)*	0.99 (0.49–1.97)
Living with elderly	Yes	78 (39.2)	1.45 (0.99–2.12)	1.43 (0.92–2.22)
	No	121 (60.1)	Ref	Ref
Family member with chronic disease	Yes	121 (60.8)	1.55 (1.07–2.25)*	1.25 (0.81–1.93)
	No	78 (39.2)	Ref	Ref
Precautionary measures in the workplace	Sufficient	29 (14.6)	Ref	Ref
	Insufficient	170 (85.4)	2.03 (1.26–3.27)*	1.89 (1.12–3.19)*
Faced stigma	Yes	131 (65.8)	2.36 (1.62–3.44)*	2.47 (1.62–3.76)*
	No	68 (34.2)	Ref	Ref
Worked overtime	Yes	107 (53.8)	1.39 (0.96–2.00)	1.31 (0. 87–1.97)
	No	92 (46.2)	Ref	Ref
Aware about government incentive	Yes	100 (50.3)	0.63 (0.44–0.91)*	0.78 (0.51–1.18)
	No	99 (49.7)	Ref	Ref
History of medication	Yes	14 (7.0)	2.54 (1.04–6.17)*	3.40 (1.31–8.81)*
	No	185 (93.0)	Ref	Ref

*Significant at *p* < 0.05

**Table 6:** Factors associated with depression among health workers (*n* = 475)

Variables	Category	Depression N (%)	Unadjusted OR (95% CI)	Adjusted OR (95% CI)
Age (years)	20–40	165 (92.7)	0.40 (0.17–0.95)*	0.33 (0.12–0.91)*
	> 40	13 (7.3)	Ref	Ref
Ethnicity	Brahmin/Chhetri	107 (60.1)	Ref	Ref
	Janajati	51 (28.7)	1.51 (0.98–2.33)	1.19 (0.74–1.93)
	Madheshi	9 (5.1)	0.87 (0.38–1.97)	1.03 (0.43–2.49)
	Others	11 (6.2)	3.53 (1.27–9.81)*	2.18 (0.73–6.57)
Profession	Doctor	40 (22.5)	0.47 (0.29–0.76)*	0.57 (0.33–0.99)*
	Nurses	78 (43.8)	1.26 (0.80–1.97)	1.25 (0.76–2.06)
	Others	60 (33.7)	Ref	Ref
Education	Intermediate and below	51 (28.7)	Ref	Ref
	Bachelor	95 (53.4)	0.44 (0.27–0.71)*	0.69 (0.41–1.16)
	Masters and above	32 (18.0)	0.38 (0.21–0.67)*	0.70 (0.35–1.40)
Living with child	Yes	53 (29.8)	1.48 (0.97–2.26)	1.19 (0.74–1.92)
	No	125 (70.2)	Ref	Ref
Precautionary measures in the workplace	Sufficient	28 (15.7)	1	Ref
	Not sufficient	150 (84.3)	1.71 (1.06–2.78)*	1.97 (1.16–3.37)*
Faced stigma	Yes	116 (65.2)	2.13 (1.45–3.12)*	2.05 (1.34–3.11)*
	No	57 (34.8)	1	Ref
Aware about government incentive	Yes	82 (46.1)	0.50 (0.34–0.72)*	0.51 (0.34–0.78)*
	No	96 (53.9)	1	Ref
History of medication	Yes	14 (7.9)	3.08 (1.27–7.51)*	3.83 (1.45–10.14)*
	No	164 (92.1)	1	Ref

*Significant at *p* < 0.05

**Table 7:** Factors associated with insomnia among health workers (*n* = 475)

Variables	Category	Insomnia N (%)	Unadjusted OR (95% CI)	Adjusted OR (95% CI)
Age (years)	20–40	108 (92.3)	0.45 (0.19–1.08)	0.45 (0.16–1.29)
	> 40	9 (7.7)	Ref	Ref
Ethnicity	Brahmin/Chhetri	70 (59.8)	Ref	Ref
	Janajati	41 (35.0)	1.90 (1.19–3.02)*	1.74 (1.04–2.91)*
	Others	6 (5.1)	0.52 (0.21–1.28)	0.40 (0.15–1.06)
Profession	Doctor	31 (26.5)	0.72 (0.42–1.25)	1.24 (0.65–2.35)
	Nurses	50 (42.7)	1.31 (0.79–2.16)	1.46 (0.82–2.60)
	Others	36 (30.8)	Ref	Ref
Education	Intermediate and below	32 (27.4)	Ref	Ref
	Bachelor	65 (55.6)	0.59 (0.36–0.99)*	0.69 (0.39–1.24)
	Masters and above	20 (17.1)	0.46 (0.24–0.88)*	0.53 (0.24–1.18)
Work experience (year)	Up to 5	71 (60.7)	0.54 (0.35–0.84)*	0.50 (0.29–0.85)*
	> 5	46 (39.3)	Ref	Ref
Affected district	Yes	94 (80.3)	1.63 (0.98–2.71)	1.55 (0.89–2.68)
	No	23 (19.7)	Ref	Ref
Faced stigma	Yes	80 (68.4)	2.26 (1.45–3.52)*	2.37 (1.46–3.84)*
	No	37 (31.6)	Ref	Ref
Aware about government incentive	Yes	58 (49.6)	0.68 (0.45–1.03)	0.66 (0.41–1.05)
	No	59 (50.4)	1	Ref
Working overtime	Yes	69 (59.0)	1.70 (1.11–2.60)*	1.53 (0.96–2.42)
	No	48 (41.0)	1	Ref
History of medication	Yes	12 (10.3)	3.98 (1.67–9.47)*	3.82 (1.52–9.62)*
	No	105 (89.7)	1	Ref

*Significant at *p* < 0.05

## Discussion

This study examined the status of anxiety, depression and insomnia symptoms among health workers in Nepal during the early phase of the COVID19 pandemic. The prevalence of anxiety (41.9%) and depression (37.5%) symptoms among health workers in this study was higher than those found in a recent study conducted among the general population during COVID-19 pandemic in Nepal, which showed that 31% of respondents reported anxiety and 34% of respondents reported depression [30]. This might be because health workers have a higher risk of acquiring COVID-19 infection in comparison to the general population, and also due to the stressful and demanding nature of the job. However, the prevalence of anxiety, depression and insomnia in this study was lower than that in health workers from China [5] where 44.6, 50.4 and 34.0% of health workers were reported to have anxiety, depression and insomnia respectively. China faced a major impact of COVID-19 and health facilities were overwhelmed with COVID-19 patients requiring hospitalisation and intensive care. The increased risk of infection and stressful environment might have contributed to higher mental health impacts among health workers in China than in Nepal [5, 8, 31]. Mental health outcomes among health workers affect their work performance and to address this, specialised mental health services are required [8, 32, 33]. The higher perceived risk and having to stay in quarantine during the epidemic might not just result in short term impacts but also lead to long term mental health consequences among health workers [34–36]. There is thus a need to focus on mental wellbeing of health workers involved in the COVID-19 response.

Our findings unveiled that a considerable proportion of health workers in Nepal faced stigma related to COVID-19. Stigma significantly affected all the psychological outcomes among health workers. Stigma among health workers, who are already vulnerable to infection due to increased exposure, might affect their concentration on work. A similar finding was observed in Italy [37] where health workers facing stigma during COVID-19 were found to have more burnout, fatigue and psychological distress. It is thus necessary to increase the morale of health workers who are stigmatised, in fear of getting infected or spreading infection to others. Importantly, the drivers and facilitators of stigma in health workers need to be understood for developing an effective response that might require extensive interventions [38, 39]. Information directed at the public should thus integrate stigma reduction among health workers as an important strategy towards COVID-19 response.

In this study, inadequate precautionary measures were significantly associated with higher odds of anxiety and depression symptoms among health workers. Lack of precautionary measures including PPE can lead to compromised working conditions, a sense of insecurity and increased exposure to infections. As a large proportion of COVID-19 cases are asymptomatic [40], lack of a proper sense of protection among health workers might increase their psychological distress and affect their mental well-being. Three out of four health workers reporting inadequate precautionary measures in the workplace in this study reflects the vulnerability of health workers in Nepal to COVID-19 infections. Studies done globally [33, 41–43] have pointed out the need to equip health workers with PPE as well as provide psychological support to increase resilience to adverse mental health outcomes. This finding should persuade the government of the urgency of arranging adequate precautionary measures for reducing mental health burden among health workers in Nepal.

Our study findings showed that nurses had higher odds of exhibiting anxiety than other health professions. This might be attributed to the higher amount of time spent by them in patient care than other health workers. A study from China also showed that nurses, compared with other health professionals, experienced more unfavourable mental health outcomes [5]. Similar findings were found during the SARS epidemic in Canada [44] where nurses experienced more psychological distress due to fear, social isolation and work stress. The mental health status of health professionals should thus be closely monitored by the employing health institutions including managing their workload, providing emotional support and responding to their personal needs.

In our study, health workers who had a history of medication for mental health problems had higher odds of exhibiting anxiety, depression and insomnia symptoms compared with those without such history. A similar finding was observed in a study conducted in China where health workers with a history of mental health problems were more likely to have anxiety, depression and stress [29]. Family and organisational support will be required for those health workers as the current pandemic might make them more vulnerable to a deterioration in their mental health conditions [45, 46].

In this study, gender, work role, marital status, and type of family, living with children, living with elderly people, having a family member with chronic disease, type of health facility, change in duties and working overtime had no significant effect on any mental health outcomes. As our study was conducted in the early phase of the pandemic when no mortality was documented and most of the cases had mild symptoms, working role might not have contributed to a significant difference in mental health outcomes. The other reason could be that health workers working in both frontline and second line might feel equally vulnerable to COVID-19 associated mental health outcomes during the early phase of pandemic. Further studies might be required to confirm these findings as the association might vary over the course of the epidemic in the country.

### Recommendations

The unprecedented challenge brought by COVID-19 pandemic is unique in Nepal and a major pressure to its health system after the 2015 Gorkha Earthquake. Health workers in Nepal are currently working under extreme pressure amidst limited health resources such as inadequate staffing, just over 3000 isolation beds and 840 ventilators (increased from 300 ventilators at the beginning of the epidemic) for the population of 29 million [3, 47, 48]. It is noteworthy that mental health has not received adequate attention from the government despite its high burden [49]. Based on the study findings, we put forward the following recommendations for improving the mental well-being of health workers in Nepal. Firstly, the reduction of stigma among health workers working in COVID-19 response should be prioritised through the mobilization of mass media and community engagement strategy. Provision should be made for living arrangements in the vicinity of health facilities if possible, which may help reduce the stigma faced by the health workers at their residence and neighborhood. This may also reduce the guilt and stress of being a potential carrier and exposing the family members to infection among health workers.. In conditions, where this is not possible,, strict measures against stigmatisation and activities such as forcing health workers to leave their rented home should be taken .. Secondly, there should be an enabling work environment with a good support system, adequate availability of PPE, proper training of health workers on management of COVID-19 and focus on incentives which boost their work morale. It is necessary to provide educational interventions for clearing of doubts of healthcare workers about COVID-19 and provide adequate logistical support to increase protection. Thirdly, personal and family support might be required especially in those who have a history of medication for mental health problems. Finally, psychological intervention with a focus on health workers should be a part of preparedness to reduce its impact not only on their well-being but also on the health system at large.

### Study limitations

The study has some limitations which need to be acknowledged. Firstly, the study was conducted during the early phase of pandemic and thus the mental health outcomes might still reflect conditions existing before the pandemic. The relative contribution of the pandemic to the increase in mental health disorders needs to be evaluated using a longitudinal study design. Secondly, there might have been the introduction of selection bias as those health workers without internet access, older health workers, and those who might have been busy in their work duties might not have participated in the study. Thirdly, there might be respondent bias as the findings were self-reported by health workers and based on a subjective scale. Importantly, the tool used in the study should be taken into consideration while reporting mental health outcomes. Although the history of mental illness and medications taken for any kind of mental illness was included in the questionnaire, specific type of mental illness was not identified, which may or may not have affected the current symptoms of anxiety, depression and insomnia. Despite limitations, this study provides early evidence on the mental health status among health workers during the COVID-19 pandemic in Nepal, which should be of interest to policymakers, health facility managers and those involved in the response to COVID-19 or anyfuture epidemic.

## Conclusions

This study reported a high prevalence of symptoms of anxiety, depression and insomnia among health workers in Nepal during the initial phase of the pandemic. More than half of the health workers faced stigma and only one out of five health workers reported precautionary measures in their workplace as sufficient. Stigma and history of medication for mental health problems was significantly associated with all the mental health outcomes while inadequate precautionary measures was associated with higher odds of having anxiety and depression symptoms. Nurses had higher odds of developing anxiety than other health workers. Improving mental wellbeing of health workers is recommended by focusing on stigma reduction, equipping health workers with protective measures, as well as ensuring personal and family support for those with a history of mental health issues.

## Supplementary information

**Additional file 1: Additional Table 1**. Anxiety and its associated factors. **Additional Table 2.** Depression and its associated factors. **Additional Table 3.** Insomnia and its associated factors

## Data Availability

All data generated during this study are included in the manuscript and the supplementary file.

## Abbreviations

AOR: Adjusted Odds Ratio
CI: Confidence interval
COVID-19: Corona virus disease 2019
HADS: Hospital Anxiety Depression Scale
ISI: Insomnia Severity Index
MERS: Middle East Respiratory Syndrome
PPE: Personal protective equipment
SARS: Severe Acute Respiratory Syndrome
SD: Standard deviation
VIF: Variance Inflation Factor
