# Supplementary material for: Mental health impacts among health workers during COVID-19 in a low resource setting: a cross-sectional survey from Nepal
Source: Global Health. 2020 Sep 25;16:89. doi: 10.1186/s12992-020-00621-z (PMC7517059; doi:10.1186/s12992-020-00621-z)
Supplement: Supplementary file 1 — Additional file 1: Additional Table 1. Anxiety and its associated factors. Additional Table 2. Depression and its associated factors. Additional Table 3. Insomnia and its associated factors [file 12992_2020_621_MOESM1_ESM.docx]

**Additional File 1**

**Mental health impacts among health workers during COVID-19 in a low resource setting: a cross-sectional survey from Nepal**

**Authors and affiliation**

**Pratik Khanal^1 ⃰^, Navin Devkota^2^, Minakshi Dahal^3^, Kiran Paudel^1^, Devavrat Joshi^2^**

^1^Institute of Medicine, Tribhuvan University, Kathmandu, Nepal

^2^National Academy for Medical Sciences, Kathmandu, Nepal

^3^Center for Research on Environment, Health and Population Activities (CREHPA), Kathmandu, Nepal

⃰ Corresponding author: [pratikkhanal@iom.edu.np](mailto:pratikkhanal@iom.edu.np)

**Additional Tables**

**Additional Table 1: Anxiety and its associated factors**

**Additional Table 2: Depression and its associated factors**

**Additional Table 3: Insomnia and its associated factors**

**Findings of bivariate analysis**

**Additional Table 1: Anxiety and its associated factors**

| **Variables** | **No Anxiety (n=276)**  **N (%)** | **Anxiety (n=199)**  **N (%)** | **X ^2^** | **P value** |
| --- | --- | --- | --- | --- |
| **Gender** |  |  |  |  |
| Male | 149 (66.2) | 76 (33.8) | 11.570 | 0.001 |
| Female | 127 (50.8) | 123 (49.2) |  |  |
| **Education** |  |  |  |  |
| Technical SLC and intermediate | 47 (50) | 47 (50) | 4.801 | 0.091 |
| Bachelors | 161 (58.1) | 116 (41.9) |  |  |
| Masters and above | 68 (65.4) | 36 (34.6) |  |  |
| **Marital status** |  |  |  |  |
| Single | 174 (58.9) | 125 (41.1) | 0.003 | 0.959 |
| Ever married | 102 (58) | 74 (42) |  |  |
| **Family type** |  |  |  |  |
| Nuclear | 180 (58.4) | 128 (41.6) | 0.41 | 0.840 |
| Joint and extended | 96 (57.5) | 71 (42.5) |  |  |
| **Living with child** |  |  |  |  |
| Yes | 66 (55.5) | 53 (44.5) | 0.456 | 0.500 |
| No | 210 (59) | 146 (41) |  |  |
| **Living with family member > 60 yrs** |  |  |  |  |
| Yes | 85 (52.1) | 78 (47.9) | 3.619 | 0.057 |
| No | 191 (61.2) | 121 (38.8) |  |  |
| **Work position** |  |  |  |  |
| Front line worker | 125 (58.4) | 89 (41.6) | 0.15 | 0.903 |
| Second line worker | 151 (57.9) | 110 (42.1) |  |  |
| **Type of health institution** |  |  |  |  |
| Primary | 34 (40.5) | 50 (59.5) | 0.084 | 0.771 |
| Secondary and tertiary | 165 (42.2) | 226 (57.8) |  |  |
| **Affected district** |  |  |  |  |
| Yes | 197 (56.3) | 153 (43.7) | 1.809 | 0.179 |
| No | 79 (63.2) | 46 (36.8) |  |  |
| **Profession** |  |  |  |  |
| Doctor | 106 (65.4) | 56 (34.6) | 21.948 | <0.001 |
| Nurse | 73 (43.7) | 94 (56.3) |  |  |
| Others | 97 (66.4) | 49 (33.6) |  |  |
| **Age** |  |  |  |  |
| 20-40 | 265 (58.5) | 188 (42.5) | 0.623 | 0.430 |
| 40-60 | 11 (50) | 11 (50) |  |  |
| **Ethnicity** |  |  |  |  |
| Brahmin/Chhetri | 203 (64.9) | 110 (35.1) | 23.257 | <0.001 |
| Janjati | 47 (40.5) | 69 (59.5) |  |  |
| Madhesi | 19 (65.5) | 10 (35.5) |  |  |
| Others | 7 (42.2) | 10 (58.8) |  |  |
| **Precautionary measure in workplace** |  |  |  |  |
| Not sufficient | 205 (54.7) | 170 (45.3) | 8.652 | 0.003 |
| Sufficient | 71 (71.0) | 29 (29.0) |  |  |
| **Family member with chronic conditions** |  |  |  |  |
| Yes | 138 (53.3) | 121 (46.7) | 5.443 | 0.020 |
| No | 138 (63.9) | 78 (36.1) |  |  |
| **Work experience** |  |  |  |  |
| Up to 5 years | 196 (58.3) | 140 (42.7) | 0.025 | 0.876 |
| >5 years | 80 (57.6) | 59 (42.4) |  |  |
| **Faced stigma** |  |  |  |  |
| Yes | 124 (48.6) | 131 (51.4) | 20.317 | <0.001 |
| No | 152 (69.1) | 68 (30.9) |  |  |
| **Aware of government incentive** |  |  |  |  |
| Yes | 170 (63) | 100 (37) | 6.064 | 0.014 |
| No | 106 (51.7) | 99 (48.3) |  |  |
| **Working overtime** |  |  |  |  |
| Yes | 126 (54.1) | 107 (45.1) | 3.048 | 0.081 |
| No | 150 (62) | 92 (38) |  |  |
| **Change in regular job duty** |  |  |  |  |
| Yes | 186 (55.7) | 148 (44.3) | 2.699 | 0.100 |
| No | 90 (63.8) | 51 (36.2) |  |  |
| **History of medication for mental health** |  |  |  |  |
| Yes | 8 (36.4) | 14 (63.6) | 4.479 | 0.034 |
| No | 268 (59.2) | 185 (40.2) |  |  |

X^2^= chi-square test; Responses of faced stigma: No and do not want to answer merged as No

**Additional Table 2: Depression and its associated factors**

| **Variables** | **No Depression (n=297)**  **N (%)** | **Depression**  **(n=178)**  **N (%)** | **X ^2^** | **P value** |
| --- | --- | --- | --- | --- |
| **Gender** |  |  |  |  |
| Male | 145 (64.4) | 80 (35.6) | 0.671 | 0.413 |
| Female | 152 (60.8) | 98 (39.2) |  |  |
| **Education** |  |  |  |  |
| Technical SLC and Intermediate | 43 (45.7) | 51 (54.3) | 14.487 | 0.001 |
| Bachelors | 182 (65.7) | 95 (34.3) |  |  |
| Masters and above | 72 (69.2) | 32 (30.8) |  |  |
| **Marital status** |  |  |  |  |
| Single | 190 (63.5) | 109 (36.5) | 0.357 | 0.550 |
| Ever married | 107 (60.8) | 69 (39.2) |  |  |
| **Family type** |  |  |  |  |
| Nuclear | 190 (61.7) | 118 (38.3) | 0.263 | 0.608 |
| Joint and extended | 107 (64.1) | 60 (35.9) |  |  |
| **Living with child** |  |  |  |  |
| Yes | 66 (55.5) | 53 (44.5) | 3.382 | 0.066 |
| No | 231 (64.9) | 125 (35.1) |  |  |
| **Living with family member > 60 yrs** |  |  |  |  |
| Yes | 100 (61.3) | 63 (38.7) | 0.147 | 0.702 |
| No | 197 (63.1) | 115 (36.9) |  |  |
| **Work position** |  |  |  |  |
| Front line worker | 136 (63.6) | 78 (36.4) | 0.059 | 0.808 |
| Second line worker | 161 (61.7) | 100 (38.3) |  |  |
| **Type of health institution** |  |  |  |  |
| Primary | 33 (39.3) | 51 (60.7) | 0.143 | 0.705 |
| Secondary and tertiary | 145 (37.1) | 246 (62.9) |  |  |
| **Affected district** |  |  |  |  |
| Yes | 217 (62.0) | 133 (38.0) | 0.157 | 0.692 |
| No | 80 (64.0) | 45 (36.0) |  |  |
| **Profession** |  |  |  |  |
| Doctor | 122 (75.3) | 40 (24.7) | 18.190 | <0.001 |
| Nurse | 89 (53.3) | 78 (46.7) |  |  |
| Others | 86 (58.9) | 60 (41.1) |  |  |
| **Age** |  |  |  |  |
| 20-40 | 288 (63.6) | 165 (36.4) | 4.601 | 0.032 |
| 40-60 | 9 (40.9) | 13 (59.1) |  |  |
| **Ethnicity** |  |  |  |  |
| Brahmin/Chhetri | 206 (65.8) | 107 (34.2) | 9.425 | 0.024 |
| Janjati | 65 (56.0) | 51 (44.0) |  |  |
| Madhesi | 20 (69.0) | 9 (31.0) |  |  |
| Others | 6 (35.3) | 11 (64.7) |  |  |
| **Precautionary measure in workplace** |  |  |  |  |
| Not sufficient | 225 (60.0) | 150 (40.0) | 4.852 | 0.028 |
| Sufficient | 72 (72.0) | 28 (28.0) |  |  |
| **Family member with chronic conditions** |  |  |  |  |
| Yes | 164 (63.3) | 95 (36.7) | 0.153 | 0.695 |
| No | 133 (61.6) | 83 (38.4) |  |  |
| **Work experience** |  |  |  |  |
| Up to 5 years | 211 (62.8) | 125 (37.2) | 0.036 | 0.849 |
| >5 years | 86 (61.9) | 53 (38.1) |  |  |
| **Faced stigma** |  |  |  |  |
| Yes | 139 (54.5) | 116 (45.5) | 15.101 | <0.001 |
| No | 158 (71.8) | 62 (28.2) |  |  |
| **Aware of government incentive** |  |  |  |  |
| Yes | 188 (69.6) | 82 (30.4) | 13.472 | <0.001 |
| No | 109 (53.2) | 96 (46.8) |  |  |
| **Working overtime** |  |  |  |  |
| Yes | 141 (60.5) | 92 (39.5) | 0.790 | 0.374 |
| No | 156 (64.5) | 86 (35.5) |  |  |
| **Change in regular job duty** |  |  |  |  |
| Yes | 207 (62.0) | 127 (38.0) | 0.145 | 0.703 |
| No | 90 (63.8) | 51 (36.2) |  |  |
| **History of medication for mental health** |  |  |  |  |
| Yes | 8 (36.4) | 14 (63.6) | 6.739 | 0.009 |
| No | 289 (63.9) | 164 (36.1) |  |  |

X^2^= chi-square test; Responses of faced stigma: No and do not want to answer merged as No

**Additional Table 3: Insomnia and its associated factors**

| **Variables** | **No insomnia (n=358)**  **N (%)** | **Insomnia**  **(n=117)**  **N (%)** | **X ^2^** | **P value** |
| --- | --- | --- | --- | --- |
| **Gender** |  |  |  |  |
| Male | 176 (78.2) | 49 (21.8) | 1.875 | 0.171 |
| Female | 182 (72.8) | 68 (27.2) |  |  |
| **Education** |  |  |  |  |
| Technical SLC and intermediate | 62 (66.0) | 32 (34.0) | 6.321 | 0.042 |
| Bachelors | 212 (76.5) | 65 (23.5) |  |  |
| Masters and above | 84 (80.8) | 20 (19.2) |  |  |
| **Marital status** |  |  |  |  |
| Single | 227 (75.9) | 72 (24.1) | 0.132 | 0.716 |
| Ever married | 131 (74.4) | 45 (25.6) |  |  |
| **Family type** |  |  |  |  |
| Nuclear | 231 (75.0) | 77 (25.0) | 0.064 | 0.800 |
| Joint and extended | 127 (76.0) | 40 (24.0) |  |  |
| **Living with child** |  |  |  |  |
| Yes | 85 (71.4) | 34 (29.6) | 1.328 | 0.249 |
| No | 273 (76.7) | 83 (23.3) |  |  |
| **Living with family member > 60 yrs** |  |  |  |  |
| Yes | 119 (73.0) | 44 (27.0) | 0.746 | 0.388 |
| No | 239 (76.6) | 73 (23.4) |  |  |
| **Work position** |  |  |  |  |
| Front line worker | 162 (71.4) | 52 (28.6) | 0.023 | 0.879 |
| Second line worker | 196 (75.1) | 65 (24.9) |  |  |
| **Type of health institution** |  |  |  |  |
| Primary | 20 (23.8) | 64 (76.2) | 0.037 | 0.847 |
| Secondary and tertiary | 97 (24.8) | 294 (75.2) |  |  |
| **Affected district** |  |  |  |  |
| Yes | 256 (73.1) | 94 (26.9) | 3.549 | 0.060 |
| No | 102 (81.6) | 23 (18.4) |  |  |
| **Profession** |  |  |  |  |
| Doctor | 131 (80.9) | 31 (19.1) | 5.171 | 0.075 |
| Nurse | 117 (70.1) | 50 (29.9) |  |  |
| Others | 110 (75.3) | 36 (24.7) |  |  |
| **Age** |  |  |  |  |
| 20-40 | 345 (76.2) | 108 (23.8) | 3.292 | 0.070 |
| 40-60 | 13 (59.1) | 9 (40.9) |  |  |
| **Ethnicity^a^** |  |  |  |  |
| Brahmin/Chhetri | 243 (77.6) | 70 (23.4) | 11.366 | 0.003 |
| Janjati | 75 (64.7) | 41 (35.3) |  |  |
| Others (including Madhesi and Dalit) | 40 (87.0) | 6 (13.0) |  |  |
| **Precautionary measure in workplace** |  |  |  |  |
| Not sufficient | 280 (74.7) | 95 (25.3) | 0.473 | 0.492 |
| Sufficient | 78 (78.0) | 22 (22.0) |  |  |
| **Family member with chronic conditions** |  |  |  |  |
| Yes | 194 (74.9) | 65 (25.1) | 0.066 | 0.797 |
| No | 164 (75.1) | 52 (24.9) |  |  |
| **Work experience** |  |  |  |  |
| Up to 5 year | 265 (78.9) | 71 (21.1) | 7.579 | 0.006 |
| >5 years | 93 (66.9) | 46 (31.1) |  |  |
| **Faced stigma** |  |  |  |  |
| Yes | 175 (68.6) | 80 (31.4) | 13.539 | 0.001 |
| No | 183 (83.2) | 37 (16.8) |  |  |
| **Aware of government incentive** |  |  |  |  |
| Yes | 212 (78.5) | 58 (21.5) | 3.344 | 0.067 |
| No | 146 (71.2) | 59 (28.8) |  |  |
| **Working overtime** |  |  |  |  |
| Yes | 164 (70.4) | 69 (29.6) | 6.115 | 0.013 |
| No | 194 (80.2) | 48 (19.8) |  |  |
| **Change in regular job duty** |  |  |  |  |
| Yes | 252 (75.4) | 82 (24.6) | 0.004 | 0.950 |
| No | 106 (75.2) | 35 (25.8) |  |  |
| **History of medication for mental health** |  |  |  |  |
| Yes | 10 (45.5) | 12 (54.5) | 11.119 | 0.001 |
| No | 348 (76.8) | 105 (23.2) |  |  |

X^2^= chi-square test; Responses of faced stigma: No and do not want to answer merged as No, ^a^Madheshi ethnic group merged into others
